# COVID-19 au Sénégal: réflexion d´un microbiologiste

**DOI:** 10.11604/pamj.supp.2020.35.2.22860

**Published:** 2020-05-12

**Authors:** Assane Dieng, Jean Baptiste Niokhor Diouf, Serigne Mbaye Lo Ndiaye

**Affiliations:** 1 Laboratoire de Bactériologie Virologie du Centre Hospitalier Universitaire le Dantec, Dakar, Sénégal; 2 Service de Pédiatrie de l´Hôpital Roi Baudouin, Dakar, Sénégal

**Keywords:** SARS CoV-2, COVID-19, virus respiratoire

## Abstract

Les maladies respiratoires particulièrement le COVID-19 constituent un problème majeur de santé publique dans le monde. Depuis mars 2020, le Sénégal a enregistré 299 cas de COVID-19 dont 183 guéris et seulement deux cas sévères. Aussi environ 20000 personnes en contact étroit avec les malades ont été testés négatifs. Ces résultats sur l´absence de cas sévère, le taux élevé de guérison et la négativité des tests chez les personnes en contact étroit avec les malades pourraient s´expliquer par un portage de coronavirus non viable ou à charge virale très faible (non détectable). En effet, certains facteurs tels que le climat, les prédispositions génétiques pourraient jouer un rôle très important sur la viabilité de SARS CoV-2. Les autres virus respiratoires tels qu´Influenza virus, VRS, rhinovirus, entérovirus, métapneumovirus, para influenza virus causant les mêmes symptômes que le SARS CoV-2, leur détection devrait être faite ensemble pour l´imputabilité de la maladie à un tel virus respiratoire. En conclusion, au Sénégal, le nombre de personnes supposées malades de COVID-19 est très faible et le taux de guérison très élevé. Ainsi, les efforts déployés contre le COVID-19 devraient être réorientés vers la prise en charge des autres pathologies prioritaires des sénégalais.

## Opinion

Depuis toujours et dans tous les pays du monde, les maladies infectieuses ont constitué l´une des premières causes de morbidité et de mortalité [1,2]. Aujourd´hui, grâce à la vaccination et les progrès de la médecine moderne, la mortalité de certaines maladies comme la variole, le tétanos, la poliomyélite, la fièvre jaune, la rougeole et la coqueluche a fortement diminué [3]. Cependant, certaines maladies respiratoires continuent de constituer un problème majeur de santé publique surtout dans les pays à ressources très limitées particulièrement en Afrique chez les enfants de moins de 5 ans, les personnes âgées ou vivant avec une maladie chronique [4,5]. En effet, les statistiques annuelles prouvent que ces infections respiratoires sont la cause de 1,9 millions de décès dans le monde dont 70% en Afrique et que les virus tels que ceux grippaux (influenza A, B, C) constituaient les principaux agents responsables [4-6]. L´incidence élevée du paludisme, de l´infection à VIH, des hépatites, des maladies rénales a fait que le fardeau des infections respiratoires en Afrique soit longtemps négligé [7]. En outre, il est établi que les virus respiratoires ne sont pas des virus pathogènes spécifiques et que bon nombre de personnes sont des porteurs asymptomatiques [8].Une étude réalisée au Sénégal avait montré que 42,1% des enfants de moins de 5 ans étaient porteurs asymptomatiques de tels virus [8].

Dans la prise en charge du COVID-19 au Sénégal, la stratégie de lutte pour endiguer l´épidémie consiste à détecter le matériel génétique du virus chez les personnes symptomatiques et asymptomatiques suspectes confinées. La charge virale de SARS CoV-2 n´est pas déterminée systématiquement. Dès lors plusieurs interrogations peuvent être posées. Les personnes déclarées malades de COVID-19 le sont-elles réellement? La recherche d´autres virus respiratoires tels qu´influenza virus ou virus respiratoire syncitial, adénovirus, rhinovirus etc. ne se révélerait-elle pas positive? Existe-t-il des arguments scientifiques plausibles démontrant l´imputabilité de ces maladies respiratoires au SARS CoV-2 uniquement et pas aux autres virus respiratoires? Au Sénégal depuis la confirmation du premier cas de COVID-19 à la date du 02 mars jusqu´au 14 avril 2020, un total de 299 patients ont été testés positifs avec seulement deux présentant une symptomatologie sévère. Parmi les 299 patients, il y´avait 183 qui étaient déclarés guéris dont un enfant de moins de deux ans et sur environ 20000 personnes qui étaient en contact étroit avec ces patients malades, les résultats d´analyse étaient négatifs. Ces résultats sur l´absence de cas sévère, le taux élevé de guérison et la négativité des tests chez les personnes de contact étroit avec les malades pourraient s´expliquer par un portage de coronavirus non viable ou à charge virale très faible (non détectable). En effet certains facteurs tels que le climat, les prédispositions génétiques pourraient jouer un rôle très important sur la viabilité de SARS CoV-2.

Pour le climat, au Sénégal l´apparition de l´épidémie a coïncidé avec la période de chaleur qui a une influence sur la viabilité des virus respiratoires [9]. Pour les facteurs de prédispositions génétiques, la négativité des tests chez les personnes contactes pourrait s´expliquer par la présence de facteurs génétiques prédisposants. Pour les malades présentant les symptômes respiratoires attribués à SARS CoV-2, les autres virus respiratoires tels qu´influenza virus, VRS, rhinovirus, entérovirus, métapneumovirus, para influenza virus et les coronavirus humains peuvent être responsables [8]. Ainsi la détection de SARS CoV-2 devrait être associée à la détection de ces virus respiratoires étant donné la ressemblance des signes cliniques que causent ces virus. Ainsi aucun argument solide n´est en faveur de l´imputabilité de ces maladies respiratoires au SARS CoV-2 d´autant qu´une étude antérieure réalisée à Dakar avait montré un portage des coronavirus humains de même que d´autres virus respiratoires chez les enfants de moins de 5 ans [8].

En conclusion, au Sénégal, le nombre de personnes supposées malades de COVID-19 est très faible. Ainsi, les efforts déployés contre le COVID-19 devraient être réorientés vers la prise en charge des autres pathologies prioritaires des sénégalais. Il y va de l´efficience dans la mise en œuvre des politiques de santé publique.

## Conflits d’intérêts

Les auteurs ne déclarent aucun conflit d’intérêts.
